# The Role of DOT1L in Normal and Malignant Hematopoiesis

**DOI:** 10.3389/fcell.2022.917125

**Published:** 2022-05-26

**Authors:** Olivia Arnold, Karina Barbosa, Aniruddha J. Deshpande, Nan Zhu

**Affiliations:** ^1^ Blood Research Institute, Versiti, Department of Cell Biology, Neurobiology and Anatomy, Medical College of Wisconsin, Milwaukee, WI, United States; ^2^ Tumor Initiation and Maintenance Program, Sanford Burnham Prebys Medical Discovery Institute, La Jolla, CA, United States

**Keywords:** DOT1l, hematopoiesis, leukemia, transcription, histone H3K79 methylation

## Abstract

Disruptor of telomeric silencing 1 (DOT1) was first identified in yeast (DOT1p) and is the sole methyltransferase responsible for histone three lysine 79 (H3K79) mono-, di-, and tri-methylation. Mammalian DOT1 (DOT1-like protein or DOT1L) has been implicated in many cellular processes, such as cell cycle progression, DNA damage response, and development. A notable developmental process reliant on DOT1L function is normal hematopoiesis, as DOT1L knockout leads to impairment in blood lineage formation. Aberrant activity of DOT1L has been implicated in hematopoietic malignancies as well, especially those with high expression of the homeobox (HOX) genes, as genetic or pharmacological DOT1L inhibition causes defects in leukemic transformation and maintenance. Recent studies have uncovered methyltransferase-independent functions and a novel mechanism of DOT1L function. Here, we summarize the roles of DOT1L in normal and malignant hematopoiesis and the potential mechanism behind DOT1L function in hematopoiesis, in light of recent discoveries.

## 1 Introduction

Disruptor of telomeric silencing 1 (DOT1 or KMT4) was first identified in yeast as a regulator of telomeric silencing, as overexpression of DOT1 decreases silencing at and around telomeric regions (Singer et al., 1998). Yeast DOT1 (DOT1p), and mammalian homolog DOT1-like (DOT1L) protein, are the sole methyltransferases (HMTs) responsible for the non-processive addition of mono-, di-, and tri-methylation to the globular portion of histone three lysine 79 (H3K79), using S-adenosylmethionine (SAM) as a methyl donor (van Leeuwen et al., 2002; Min et al., 2003; Frederiks et al., 2008; Feng et al., 2010). Of these marks, H3K79me2/3 are found in gene bodies and believed to be associated with transcription elongation (Barry et al., 2009; Godfrey et al., 2019). DOT1 lacks a SU(var), Enhancer of Zeste, and Trithorax (SET) domain, distinguishing it from other lysine methyltransferases (van Leeuwen et al., 2002; Farooq et al., 2016).

Studies show DOT1L is important for cellular processes including cell cycle progression, DNA damage repair, and transcriptional regulation (Reviewed in (Kim et al., 2014; Wood et al., 2018)). DOT1L plays roles in developmental processes and its deletion in mice is embryonic lethal (Jones et al., 2008; Feng et al., 2010). DOT1L is important for developmental hematopoiesis, in which pluripotent hematopoietic stem cells differentiate into multipotent progenitors, followed by terminal differentiation into various mature cell types. Given the similarities in the hierarchical development of normal and malignant hematopoiesis, studies have probed the function of DOT1L in both contexts. Research in human and mouse models illustrates a role of DOT1L in hematopoietic development (Feng et al., 2010; Nguyen et al., 2011a; Bernt et al., 2011; Jo et al., 2011). Aberrant DOT1L activity is found in various hematopoietic malignancies including AML with KMT2A (MLL) gene rearrangements (Okada et al., 2005; Bernt et al., 2011; Jo et al., 2011), partial tandem duplications (Kühn et al., 2015), NPM1 mutations (Kühn et al., 2016), MLLT10 (AF10) gene fusions (Chen et al., 2013), and NUP98-rearranged AML (Deshpande et al., 2014). The exact mechanism of function of DOT1L in these contexts remains elusive, however there is compelling evidence showing histone methylation by DOT1L activates subsets of genes involved in hematopoietic stem cell (HSC) development that is coopted by leukemia cells in various AML subtypes. This includes homeobox (HOXA or HOXB cluster) genes, and the three-amino acid loop-extension (TALE) HOX co-factor MEIS1. While the current body of work has focused on the enzymatic role of DOT1L accounting for its function, recent studies have explored methyltransferase-independent mechanisms. In this review, we summarize what is known about DOT1L in normal and malignant hematopoiesis as well as new mechanistic insights into its function.

## 2 Normal Hematopoiesis

DOT1L constitutive knockout (KO) is embryonic-lethal in mice (Jones et al., 2008; Feng et al., 2010). By embryonic day 9.5 (E9.5), DOT1L KO embryos displayed heart dilation, stunted tails, defective yolk sac angiogenesis, and were overall smaller than wild type (WT) controls. At E10.5, viable KO embryos fell below the expected ratios and no surviving KO embryos were observed by E13.5. DOT1L KO cells derived from blastocysts showed aneuploidy, telomere elongation, and proliferation defects (Jones et al., 2008). Feng et al. found a similar embryonic lethality phenotype of DOT1L KO mice, likely due to severe anemia and associated defective yolk sac angiogenesis. Further, primitive and definitive yolk sac erythroid progenitors displayed decreased colony formation in CFU assays (Feng et al., 2010). The E10.5 yolk sac showed an increased proportion of cells in G0/G1 with a concomitant decrease in S and G2/M phases of the cell cycle and increased apoptosis compared to WT controls (Feng et al., 2010). Mechanistically, DOT1L loss resulted in decreased GATA2 and increased PU.1 expression, accounting for erythropoiesis defects (Feng et al., 2010).

A constitutive DOT1L methyltransferase mutant (DOT1L-MM N241D) mouse model was recently characterized, showing potential non-enzymatic roles of DOT1L in hematopoiesis during early development. Similar to DOT1L KO mice, DOT1L-MM embryos died before E13.5 (Malcom et al., 2021), but showed no anemia or defective angiogenesis in the yolk sac or the aorta-gonad-mesonephros region (Malcom et al., 2021). Colony formation by primitive and definitive yolk sac erythroid progenitors was not impaired, as similar colony numbers were observed in DOT1L-MM and WT, although the definitive yolk sac progenitor-derived colonies were smaller in DOT1L-MMs (Malcom et al., 2021). The formation of myeloid or mixed colonies by definitive yolk sac progenitors (E10.5) was also reduced (Malcom et al., 2021). These data suggest a methyltransferase-independent function is responsible for DOT1L’s role in embryonic erythropoiesis (Malcom et al., 2021). Consistent with the phenotypic differences between DOT1L KO and DOT1L-MM, gene expression analysis of extensively self-renewing erythroblasts showed overlapping and distinct sets of differentially expressed genes. Signatures affected in both models included cell proliferation, cell cycle regulation, and HSC differentiation, including Hoxa9 (Borosha et al., 2022).

The role of DOT1L in adult hematopoiesis has also been extensively studied. In a constitutive VavCre knockout model, Dot1l deletion by E10.5 resulted in litters born at expected frequencies, with normal body and organ weight. However, loss of DOT1L in young mice (3–6 weeks) resulted in anemia, neutropenia, lymphopenia, and reduced BM cellularity with significant reductions in HSPC compartments. Older mice showed partial chimerism due to non-deleted clones (Bernt et al., 2011). Using the inducible whole-body knockout mouse model, Dot1l^f/f^ Cre-ER, postnatal Dot1l deletion resulted in pancytopenia, BM hypocellularity, and reductions of HSPC and mature cells in all lineages during steady-state hematopoiesis (Nguyen et al., 2011a; Jo et al., 2011). These effects were cell-autonomous, as Dot1l KO cells were outcompeted in BM transplantations (Nguyen et al., 2011a; Jo et al., 2011). Repeated tamoxifen injection to maintain deletion led to lethality 2–3 months post-induction with severe hematopoiesis defects (Jo et al., 2011). One study noticed anemia and bleeding in multiple organs upon Dot1l deletion (Nguyen et al., 2011a). Similar effects on steady-state hematopoiesis, namely BM hypocellularity and decreases in HSPC compartments, were observed in the Dot1l^f/f^ MxCre model upon polyinosinic:polycytidylic acid-mediated DOT1L deletion in hematopoietic cells (Grigsby et al., 2021). Overall, these studies point to an essential role of DOT1L in adult normal hematopoiesis by affecting HSC self-renewal and differentiation. It is known that Dot1l regulates the homeobox transcription factors Hoxa9 and Meis1, as its loss results in decreased H3K79 methylation and expression of both genes (Deshpande et al., 2014). Based on the known function of DOT1L (Nguyen and Zhang, 2011), and data from embryonic hematopoiesis, it is likely that DOT1L loss affects cell cycle progress and/or apoptosis. Indeed, DOT1L deletion led to global loss of H3K79 methylation (Nguyen and Zhang, 2011; Deshpande et al., 2014), and experimental evidence shows that methyltransferase activity is required for normal adult hematopoiesis. Grigsby et al. utilized methyltransferase mutants in which the SAM-binding domain was mutated in rescue experiments in a normal hematopoiesis study. They found that WT, but not the enzymatic dead mutant, can rescue Dot1l KO HSPCs expanded by NUP98-HOXD10HD in transplant models (Grigsby et al., 2021), suggesting that DOT1L methyltransferase activity is required in this context.

### 2.2 Malignant Hematopoiesis

**FIGURE 1:**
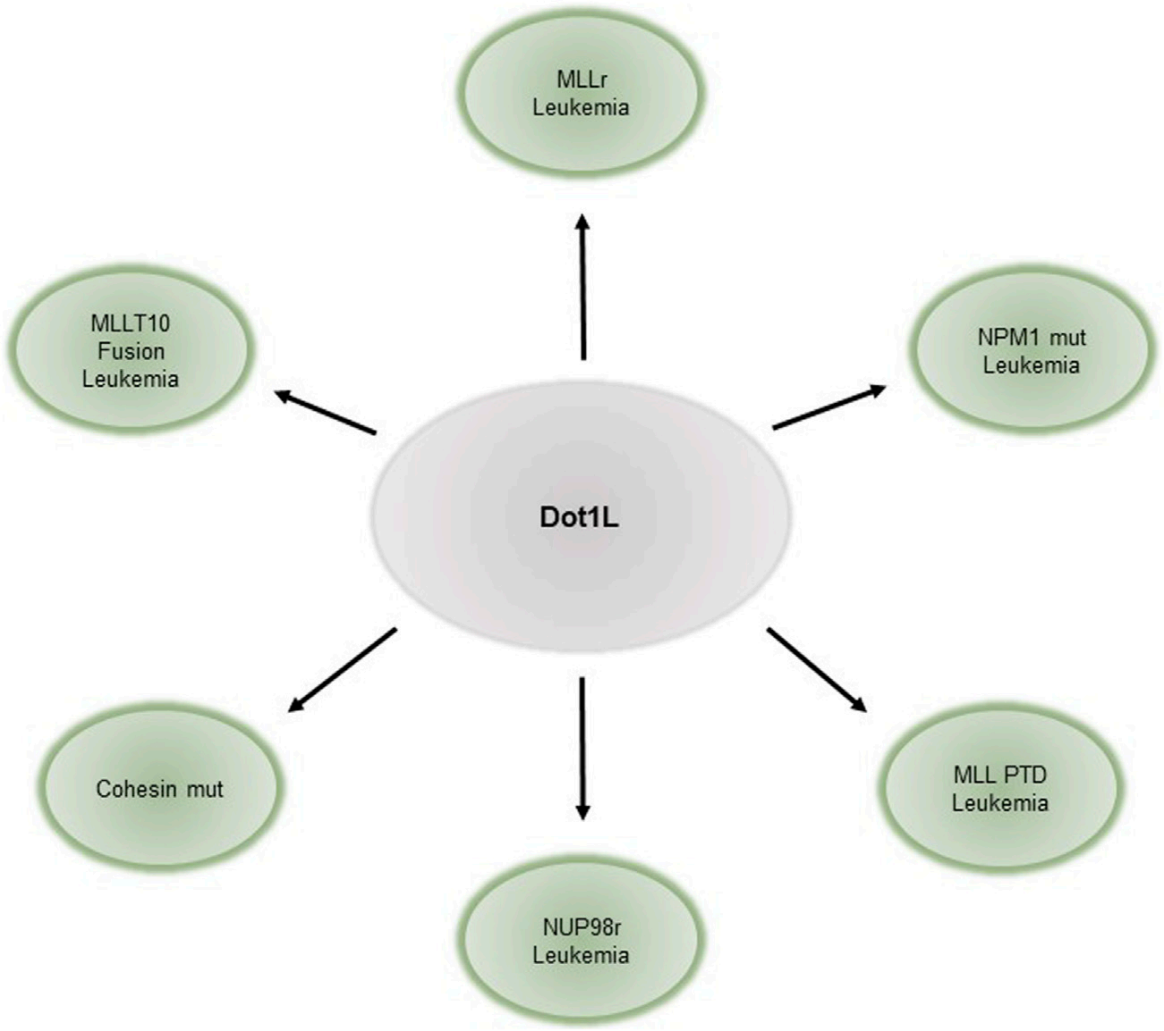
Requirement of DOT1L in hematopoietic malignancies.

DOT1L has also been implicated in hematopoietic malignancies, especially in leukemias harboring Mixed Lineage Leukemia gene (MLL) rearrangements (MLLr, reviewed elsewhere Nguyen and Zhang, 2011; Chen and Armstrong, 2015; Wang et al., 2016). Translocations of MLL fuse its N- terminus to one of over 60 different fusion-partner genes (Krivtsov and Armstrong, 2007; Meyer et al., 2009; Muntean and Hess, 2012). These fusions are found in approximately 70% of infant leukemias, and 10% of leukemias in older individuals. Some of the most common fusion-partners of MLL are members of various elongation complexes and the Dot1 complex such as AFF1/AF4, MLLT3/AF9, MLLT10/AF10, MLLT1/ENL (reviewed in (Nguyen and Zhang, 2011; Deshpande et al., 2012)). The resulting onco-fusion proteins recruit DOT1L to target gene promoters, resulting in abnormally high levels of H3K79me (Krivtsov et al., 2017) and aberrant gene activation (Jo et al., 2011). Canonical targets of these onco-fusion proteins include HOXA9 and MEIS1. Genetic or pharmacological inhibition of DOT1L leads to downregulation of target genes and impaired proliferation, cell cycle, and survival of leukemia cells in leukemias driven by MLL fusion (Okada et al., 2005; Chang et al., 2010; Bernt et al., 2011; Nguyen et al., 2011b; Jo et al., 2011; Deshpande et al., 2013). Similarly, leukemias bearing AF10 gene fusions require DOT1L for leukemia initiation and maintenance, and DOT1L loss leads to decreased expression of downstream fusion targets (Chen et al., 2013). DOT1L, however, is dispensable for BCR-ABL, E2A-HLF, E2A-PBX2 leukemias, and leukemia generated by ectopic retroviral overexpression of HOXA9/MEIS1 (Chang et al., 2010; Jo et al., 2011; Richter et al., 2021). Thus, the requirement of DOT1L is context-specific and not a general requirement for cell proliferation. Leukemias susceptible to DOT1L loss are summarized in Figure 1.

The prominent role of DOT1L in MLLr leukemia has led to the development of targeted therapies. DOT1L methyltransferase inhibitors increase differentiation of MLLr leukemia cells and decrease proliferation, global H3K79me, and expression of HOXA9 and MEIS1 (Daigle et al., 2011). Clinical trials of the DOT1L inhibitor Pinometostat showed it is well-tolerated, however, the efficacy is modest as a single agent (Stein et al., 2018). Albeit limited, its efficacy in MLLr leukemia may hold promise for use in combination therapies (Stein et al., 2018). Pinomestostat was also effective in NPM1 mutant leukemia and can lower FLT3, MEIS1, and HOXB cluster gene expression (Kühn et al., 2016). Similarly, DOT1L inhibition blocked cohesin loss, induced abnormal self-renewal, and caused aberrant HOXA9 expression in *Rad21* knockdown as well as *Smc3* heterozygous cells in mouse (Heimbruch et al., 2021). Beyond HOX and MEIS1 gene regulation, DOT1L has been shown to transcriptionally regulate FLT3 and its downstream pathways. In MLLr cell lines carrying FLT3-ITD, an activating FLT3 mutation, the increased susceptibility of the cells to DOT1L inhibitor at a dose without any effect on HOXA9/MEIS1 expression is attributed to FLT3 regulation (Richter et al., 2021). Consistently, non-MLLr cell lines with FLT3-ITD mutations were susceptible to DOT1L inhibition (Richter et al., 2021). In addition to DOT1L’s methyltransferase activity, studies have shown that loss of Dot1 complex (DotCom, composed of AF9, AF10, ENL and AF17) components AF10 (Deshpande et al., 2014), and ENL (Wan et al., 2017) have a similar effect on leukemia cells as loss of DOT1L. Similarly, the DOT1L and AF9 interaction has been shown to be important for leukemogenesis (Shen et al., 2013; Kuntimaddi et al., 2015; Grigsby et al., 2021). Studies of methyltransferase mutant, AF9-binding disrupted mutant, and wild type DOT1L models *in vivo* showed loss of DOT1L-AF9 was sufficient to inhibit leukemia cell growth and increase their differentiation to similar levels observed with DOT1L enzyme-dead mutant (Shen et al., 2013; Kuntimaddi et al., 2015; Grigsby et al., 2021). Studies also suggest the cooperation of the AEP elongation complex (AF4, AF5q31, ENL and p-TEFb) and DotCom in the activation and maintenance of aberrant gene expression, and is critical in MLLr cell transformation, providing a rationale for combinatory targeting of DOT1L and Menin, which targets MLL fusion to chromatin to eradicate leukemia cells (Kühn et al., 2016; Dafflon et al., 2017; Okuda et al., 2017; Olsen et al., 2022). Overall, recent research has expanded the utility of DOT1L inhibition in leukemias outside MLLr and points to combination therapy involving DOT1L as promising in development of novel targeted therapies.

### 2.3 DOT1L in Transcription Regulation

#### 2.3.1 Elongation and Initiation

**FIGURE 2:**
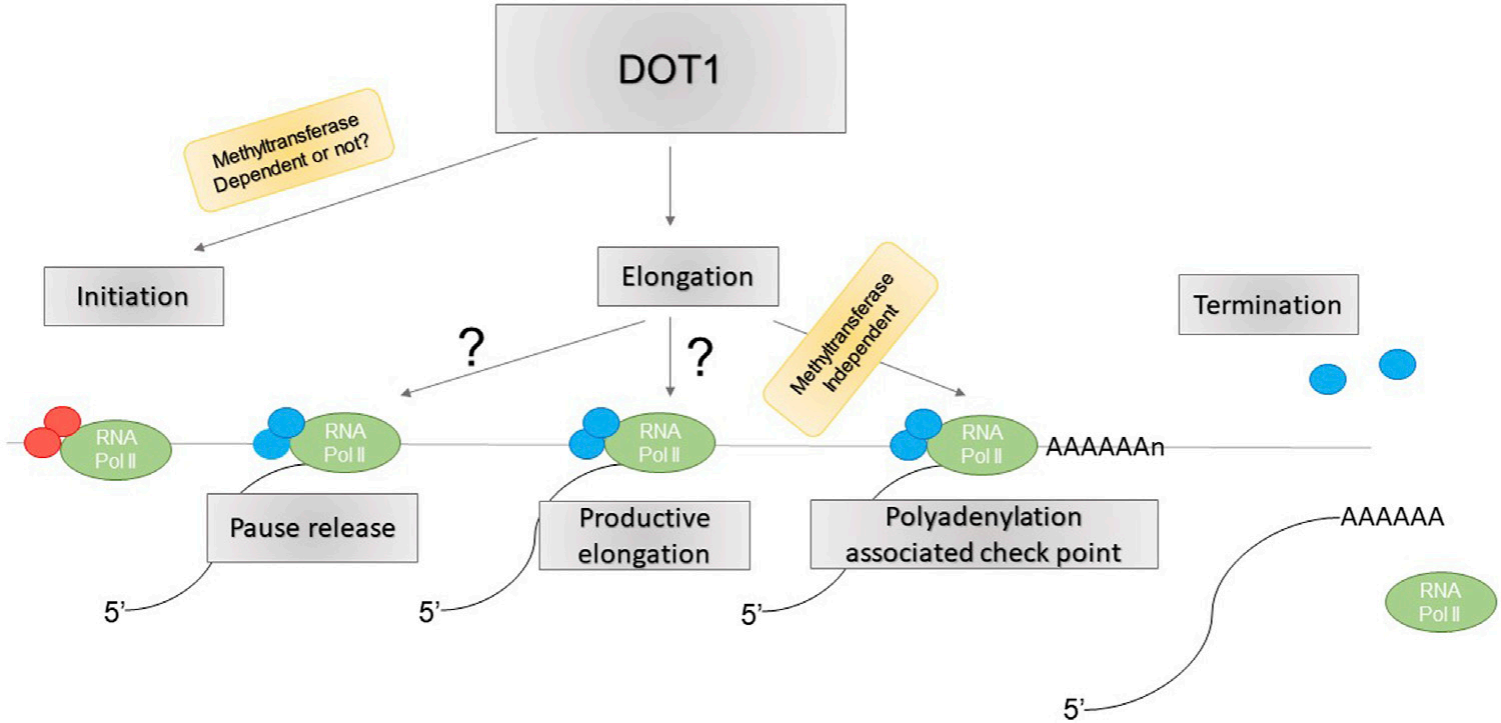
Role of DOT1L in transcription regulation.

DOT1L is believed to be involved in transcription elongation (reviewed in (Nguyen and Zhang, 2011; Wood et al., 2018). However, a recent study suggests this role is minimal, and rather, DOT1L mediates transcription initiation. Loss of DOT1L led to reduced Pol II chromatin association globally and direct measurements of transcription elongation showed no difference between DOT1L KD and controls (Wu et al., 2021). These included traveling ratio of Pol II, measured by ChIP-seq or PRO-seq, and elongation rate, measured by 4sUDRB-seq (Wu et al., 2021). Instead, recruitment of general transcription factors (GTFs) TBP, TFIIA, and TFIIB required for transcription initiation to gene promoters was significantly reduced upon DOT1L KD (Wu et al., 2021). The physical interaction between DOT1L and these GTFs, and its ability to recruit TFIID to chromatin, may underlie their recruitment by DOT1L (Wu et al., 2021). Similarly, Cao et al. showed that inhibition of super elongation complex (SEC) activity causes accumulation of proximal RNA Pol II as a result of impaired RNA Pol II pause release, necessary for transition into productive elongation (Cao et al., 2020). However, DOT1L deletion coupled with SEC inhibition showed a similar phenotype as SEC inhibition alone, suggesting DOT1L may not be required for RNA Pol II pause release (Cao et al., 2020). Rather, DOT1L deletion further exacerbated defects in Pol II accumulation near transcription termination sites upon SEC inhibition (Cao et al., 2020). Interestingly, this effect was independent of DOT1L methyltransferase activity, as cells containing catalytically inactive DOT1L did not show such defects (Cao et al., 2020). Together, these studies point to regulation of transcription initiation, in addition to elongation, and Poly(A) associated elongation checkpoint, not pause release, as potential mechanisms for DOT1L function in gene transcription (Figure 2), although the detailed mechanisms and regulatory specificity remain unknown.

#### 2.3.2 DOT1 Interactions With Ubiquitinated Histone H2B

Methylation of H3K79 by DOT1L depends on H2B K120 ubiquitination (H2Bub) and such crosstalk is conserved from yeast to metazoan. Recent cryo-electron microscopy (Cryo-EM) studies of DOT1L bound to ubiquitinated nucleosomes showed that DOT1L interacts with H2Bub through a C-terminal hydrophobic helix. Additional contact is made with an arginine anchor within DOT1L and an H2A/H2B acidic patch in both poised and active DOT1L complexes. In the active complex, binding of H4 tail to a cleft in DOT1L positions the catalytic center above H3K79 and induces conformation changes in H3 to reposition the inaccessible H3K79, inserting the side chain into the active site (Anderson et al., 2019; Jang et al., 2019; Valencia-Sánchez et al., 2019; Worden et al., 2019; Yao et al., 2019). Further, yeast Dot1 was shown to promote H2Bub and interact with the SAGA complex, which deubiquitinates H2B (van Welsem et al., 2018). Interestingly, this function is independent of the methyltransferase activity of Dot1 (van Welsem et al., 2018). Similarly, DOT1L KO leads to increased chromatin association of SAGA complex and decreased H2Bub, suggesting DOT1L promotes H2Bub by limiting SAGA recruitment (Wu et al., 2021). This study did not address whether DOT1L enzymatic activity is required for promoting H2Bub in mammalian cells, as it does in yeast.

#### 2.3.3 A Role for DOT1L in Enhancer Regulation

Beyond their role in transcription elongation, H3K79me2/3 marks have been found in functionally active enhancers (Bonn et al., 2012; Markenscoff-Papadimitriou et al., 2014; Gilan et al., 2016). Godfrey et al. (2019) showed that H3K79me2/3 are found on a subset of active enhancers, dubbed KEEs (H3K79me2/3 enhancer elements) (Godfrey et al., 2019). KEEs are functional enhancers associated with higher gene expression and increased enhancer-promoter interaction (Godfrey et al., 2019). Loss of H3K79me2/3 upon DOT1L inhibition leads to reduced chromatin accessibility and H3K27ac, but not H3K4me1, typically associated with enhancer elements and TF binding in MLL-AF4 cells (Godfrey et al., 2019).

## 3 Conclusion and Future Perspectives

DOT1L has been implicated in many important processes including cell cycle, transcription regulation, DNA damage repair, and general development. In normal hematopoiesis, DOT1L is required for both embryonic and adult hematopoiesis by regulating genes important in HSC differentiation and proliferation, such as HOX/MEIS and FLT3. Recent findings show that DOT1L may function in a methyltransferase-independent manner in primitive erythropoiesis (Malcom et al., 2021), in a yet unclear mechanism. Conversely, Grigsby et al. showed that DOT1L’s function in adult hematopoiesis seems to depend on its methyltransferase activity. However, they utilized HSPCs expanded by NUP98-HOXD10 to test if methyltransferase mutants could rescue the effects of DOT1L (Grigsby et al., 2021), which raises concerns about the physiological relevance of such finding. Thus, an assessment of conditional DOT1L enzymatic-dead knock-in mouse models is needed to examine the enzymatic contribution of DOT1L in adult hematopoiesis.

Aberrant activity of DOT1L is implicated in many hematopoietic malignancies, particularly MLL-rearranged leukemias. The canonical target genes of DOT1L in leukemia are similar to those in normal hematopoiesis, including *HOX* and *FLT3* genes. Recent research has provided a rationale to target DOT1L outside of MLLr leukemia, including in NPM1 (Kühn et al., 2016) and cohesin-mutated leukemias (Heimbruch et al., 2021). Researchers have also laid the basis for targeting DOT1L complex formation or its members (Shen et al., 2013; Deshpande et al., 2014; Kuntimaddi et al., 2015; Kühn et al., 2016; Dafflon et al., 2017; Okuda et al., 2017; Wan et al., 2017; Grigsby et al., 2021; Olsen et al., 2022). Given the role of DOT1L and other elongation complexes in the aberrant transcription programs of MLL fusions, combinatorial targeting of key players such as DOT1L and MENIN, has shown promise compared to the use of single inhibitor agents. This is especially relevant since DOT1L inhibitors as single-agent therapeutics lack efficacy. An important open question is whether enzyme-independent functions are important for DOT1L’s role in leukemogenesis. Given that the phenotypes for DOT1L pharmacological and genetic inhibition display divergent kinetics, namely H3K79me loss and target gene expression changes, it is possible that a non-enzymatic dependent function is required. If so, strategies targeting the entire protein rather than the enzymatic function should be considered in the development of targeted therapeutics.

Recent reports have advanced our understanding of the molecular mechanism of DOT1L function. Two studies have shown that DOT1L does not play a major role in transcription elongation. Instead, one points to its role in transcription termination checkpoint control, which is independent of its enzymatic function (Cao et al., 2020), and the other addresses its role in transcription initiation through GTF recruitment (Wu et al., 2021). Further, DOT1L limits SAGA complex association with chromatin, providing a mechanism for interlinked H2Bub and H3K79me2/3 levels (Wu et al., 2021). Finally, KEE enhancers containing the H3K79me2/3 have been shown to be functional enhancers (Godfrey et al., 2019). Both SAGA complex and enhancers play a role in transcription initiation, perhaps lending support to the regulation of transcription initiation by DOT1L. Finally, given the involvement of condensate formation in transcription regulation and the existence of separate condensates for transcription initiation and elongation (Guo et al., 2019), it will be interesting to inquire a potential regulatory layer for DOT1L in these structures.
